# Insights into the early transcriptomic response against watermelon mosaic virus in melon

**DOI:** 10.1186/s12870-024-04745-x

**Published:** 2024-01-20

**Authors:** María López-Martín, Javier Montero-Pau, Guillem Ylla, María Luisa Gómez-Guillamón, Belén Picó, Ana Pérez-de-Castro

**Affiliations:** 1 COMAV, Instituto de Conservación y Mejora de la Agrodiversidad Valenciana, Universitat Politècnica de València, Cno. de Vera, s/n, 46022 València, Spain; 2 Instituto Cavanilles de biodiversidad y la biología evolutiva (ICBIBE), Universidad de Valencia, C/ del Catedrátic José Beltrán Martínez, 2, 46980 Paterna, Spain; 3 Laboratory of Bioinformatics and Genome Biology, Faculty of Biochemistry, Biophysics and Biotechnology, Jagiellonian University, 30-387 Kraków, Poland; 4 Instituto de Hortofruticultura Subtropical y Mediterránea La Mayora, CSIC-UMA, Avda. Dr. Wienberg s/n, 29750 Málaga, Spain

**Keywords:** WMV, Melon, TGR-1551, RNA-seq, WGCNA, SNPeff, Mediator complex, MED33

## Abstract

**Background:**

Watermelon mosaic virus (WMV) is one of the most prevalent viruses affecting melon worldwide. Recessive resistance to WMV in melon has previously been reported in the African accession TGR-1551. Moreover, the genomic regions associated to the resistance have also been described. Nevertheless, the transcriptomic response that might infer the resistance to this potyvirus has not been explored.

**Results:**

We have performed a comparative transcriptomic analysis using mock and WMV-inoculated plants of the susceptible cultivar “Bola de oro” (BO) and a resistant RIL (Recombinant inbred line) derived from the initial cross between “TGR-1551” and BO. In total, 616 genes were identified as differentially expressed and the weighted gene co-expression network analysis (WGCNA) detected 19 gene clusters (GCs), of which 7 were differentially expressed for the genotype x treatment interaction term. SNPs with a predicted high impact on the protein function were detected within the coding regions of most of the detected DEGs. Moreover, 3 and 16 DEGs were detected within the QTL regions previously described in chromosomes 11 and 5, respectively. In addition to these two specific genomic regions, we also observde large transcriptomic changes from genes spread across the genome in the resistant plants in response to the virus infection. This early response against WMV implied genes involved in plant-pathogen interaction, plant hormone signal transduction, the MAPK signaling pathway or ubiquitin mediated proteolysis, in detriment to the photosynthetic and basal metabolites pathways. Moreover, the gene MELO3C021395, which coded a mediator of RNA polymerase II transcription subunit 33A (*MED33A*), has been proposed as the candidate gene located on chromosome 11 conferring resistance to WMV.

**Conclusions:**

The comparative transcriptomic analysis presented here showed that, even though the resistance to WMV in TGR-1551 has a recessive nature, it triggers an active defense response at a transcriptomic level, which involves broad-spectrum resistance mechanisms. Thus, this study represents a step forward on our understanding of the mechanisms underlaying WMV resistance in melon. In addition, it sheds light into a broader topic on the mechanisms of recessive resistances.

**Supplementary Information:**

The online version contains supplementary material available at 10.1186/s12870-024-04745-x.

## Background

Melon (*Cucumis melo* L.) is one of the main cucurbit crops cultivated worldwide. Its selection in different countries of the Mediterranean basin and Eastern Asia has led to a great phenotypic and genotypic variability [1]. This diversity is commonly used as a source of alleles in breeding programs to introgress different characters of interest in elite cultivars. Among these programs, those aimed to produce virus-resistant varieties are of particular importance. Viruses in the main melon producing areas have a great yield-limiting potential and are a major economic concern for growers [2]. Different studies carried out recently in Europe [3–5], Asia [6–8] and America [9, 10] indicate that the potyvirus Watermelon mosaic virus (WMV) is the most prevalent virus in cucurbits fields. WMV infection in melon leads to a severe symptomatology, that includes chlorosis, mosaic, leaf distortion, lead tip stunting, as well as the stop of plant growth, which results in yield reduction and fruit quality loss. Moreover, phylogenetic studies have shown that WMV is constantly evolving due to recombination and mutation events, leading to more virulent strains [5, 9, 11].

Since potyviruses are transmitted in a non-persistent manner, cultural practices and the use of pesticides are useless to control this virus. Several melon accessions have been described as tolerant to WMV [12–15], but only two accessions, PI 414723 and TGR-1551, have been found to be resistant to WMV [16, 17]. The phenotyping and genotyping by sequencing (GBS) of a RIL (Recombinant inbred lines) population derived from the cross between TGR-1551 and the susceptible cultivar “Bola de oro” (BO) allowed to map a major resistance QTL, named *wmv*^*1551*^, on a 130 kb interval in chromosome 11. Moreover, a minor QTL with a significant effect on heterozygous plants for the introgression in chromosome 11 was also mapped to a 700 kb region on chromosome 5 [18].

Functional characterization of the genes located in the candidate resistance regions is required to understand the molecular resistance mechanisms against WMV in melon. Usually, recessive resistances against viruses are caused by the loss or the mutation of a susceptibility factor, necessary for the virus to complete its life cycle [19]. Actually, a *Vacuolar protein sorting 4* located out of the described candidate region on chromosome 11 was proposed as a susceptibility factor to WMV infections [20]. Nevertheless, a microarray expression analysis of 17.443 unigenes after the inoculation of TGR-1551 and a susceptible variety with WMV, revealed a great transcriptomic remodeling [21]. Moreover, the analysis of the small RNAome by high-throughput pyrosequencing after the inoculation with WMV suggests that mechanisms of RNA silencing could also be implied in the resistance [22].

The cheapening of new generation sequencing (NGS) technologies has popularized the use of RNA-seq technology to detect differentially expressed genes (DEGs) in cucurbits plants during viral infections [23–27]. Since RNA-seq provides an enhanced transcriptome coverage, with greater accuracy than microarrays, it is more useful to find new transcription features, identify DEGs and alternative gene splicing. Nevertheless, to our knowledge, there are currently no studies using RNA-seq technology to detect differentially expressed genes related to the defense response against WMV. Moreover, to date, as far as we know, there is only one RNA-seq assay has been conducted to understand the resistance mechanisms against viruses in melon [25].

In this study, we provide new insights into the genetic and transcriptomic basis of WMV resistance in melon, by performing and exhaustive comparative transcriptomic analysis between resistant and susceptible melon lines. We used mock-inoculated and WMV infected melon plants of the resistant RIL-10-3, derived from the accession TGR-1551, and its susceptible parental line “Bola de oro” at 3 days after treatment to identify the transcriptomic changes implied in the early resistance response to the virus.

## Results

### Assessment of RIL-10-3 and BO response to watermelon mosaic virus infection

**Fig. 1:**
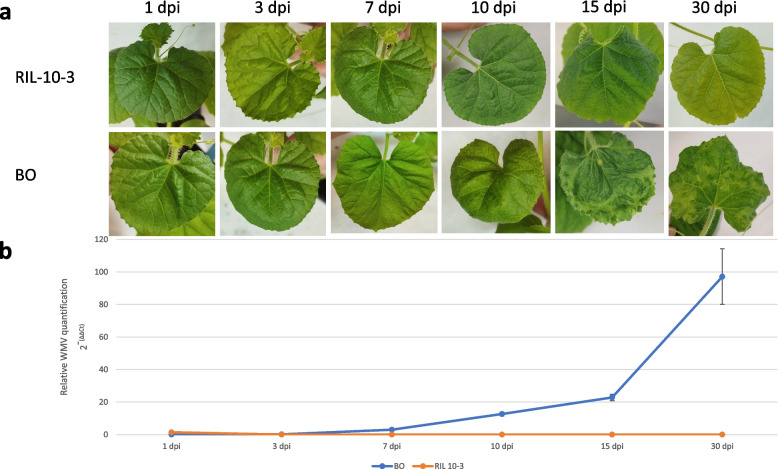
Assessment of RIL-10-3 and BO response to WMV infection in the first total expanded apical leaf of each plant. **a** Temporal evolution of symptomatology. **b** Mean of viral titers in RIL-10-3 and BO measured by RT-qPCR

All the 24 RIL-10-3 plants inoculated with WMV remained symptomless during the assay (Fig. 1a), while 24/24 inoculated plants of the susceptible cultivar BO started to show mild-severe symptoms (score 2–3) at 10 days post-inoculation (dpi). The symptomatology increased over time and at 15 and 30 dpi severe mosaic and leaf curling was observed in BO plants (Fig. 1a). Moreover, those plants sampled without symptoms at early infection stages were allowed to regrow and it was possible to identify symptoms in all of them at 30 dpi.

The accumulation of WMV in both RIL-10-3 and BO inoculated plants was confirmed by RT-qPCR. Significant differences (*p*-value< 0.05) were observed between both lines over time. The initial viral accumulation of RIL-10-3 was higher at 1 dpi but it remained at low levels during the course of the experiment, whereas the viral load of BO samples started to rise significantly at 7 dpi, achieving its higher accumulation at 30 dpi (Fig. 1b). These results showed that WMV was able to infect both susceptible and resistant genotypes, but its movement or replication was inhibited in the resistant RIL-10-3, whereas in BO the infection was systemic after 7 dpi. These results suggested that the resistance response is activated in the resistant genotype before the infection becomes systemic. Given that at 7dpi the infection was already systemic, we collected samples just in the previous time point, at 3 dpi, to be analyzed by RNA-seq. Moreover, 0 dpi samples were also included as controls.

### RNA sequencing and mapping

A total of 18 libraries were sequenced, producing on average almost 43.7 million reads per sample. After excluding short and low-quality reads, between 20.3 and 29.7 million reads per sample were obtained. The clean reads were mapped to the melon reference genome (v.4.0) [28] with the STAR algorithm [29]. The percentage of uniquely mapped reads ranged from 80.65 to 94.62% (Table 1). These transcriptomes were further analyzed to compare the interaction between genotype (resistant or susceptible) and treatment (virus or mock-inoculated).

**Table 1:** Summary of the RNA-seq experiment design, number of raw reads, number of clean reads (percentage of raw reads after cleaning), total reads mapped to the reference genome (percentage of clean reads that have mapped to the reference genome) and number of reads uniquely mapped (percentage of reads uniquely mapped relative to the number of mapped reads) (dpi: days post-inoculation)

Sample name	Phenotype	Inoculation treatment	Time point dpi	Replicate	Raw reads	Cleaned reads (%)	Mapped reads (%)	Uniqueli mapped reads (%)
RIL-10-3-M3dpi_1	Resistant	Mock-inoculated	3	1	41,429,310	20,676,828 (49.91%)	17,808,540 (42.99%)	15,343,838 (86.16%)
RIL-10-3-M3dpi_2	Resistant	Mock-inoculated	3	2	42,112,204	21,017,363 (49,91%)	19,272,192 (45.76%)	17,672,600 (91.70%)
RIL-10-3-M3dpi_3	Resistant	Mock-inoculated	3	3	42,695,048	21,304,504 (49.9%)	17,246,449 (40.39%)	13,961,000 (80.95%)
RIL-10-3-W3dpi_1	Resistant	WMV-inoculated	3	1	41,583,644	20,750,266 (49.9%)	19,102,285 (45.94%)	17,585,563 (92.06%)
RIL-10-3-W3dpi_2	Resistant	WMV-inoculated	3	2	43,083,818	21,508,031 (49.92%)	20,350,015 (47.23%)	19,255,184 (94.62%)
RIL-10-3-W3dpi_3	Resistant	WMV-inoculated	3	3	43,009,278	21,463,096 (49.9%)	19,980,658 (46.46%)	18,599,994 (93.09%)
RIL-10-3-0dpi_1	Resistant	Uninoculated	0	1	40,318,008	20,124,842 (49.92%)	16,545,112 (41.04%)	13,601,736 (82.21%)
RIL-10-3-0dpi_2	Resistant	Uninoculated	0	2	43,093,538	21,504,205 (49.9%)	18,964,458 (44.01%)	16,724,755 (88.19%)
RIL-10-3-0dpi_3	Resistant	Uninoculated	0	3	59,630,926	29,763,672 (49.91%)	24,909,634 (41.77%)	20,846,872 (83.69%)
BO-M3dpi_1	Susceptible	Mock-inoculated	3	1	42,827,098	21,369,863 (49.9%)	17,234,566 (40.24%)	13,899,677 (80.65%)
BO-M3dpi_2	Susceptible	Mock-inoculated	3	2	44,335,964	22,126,097 (49.91%)	20,783,260 (46.88%)	19,521,716 (93.93%)
BO-M3dpi_3	Susceptible	Mock-inoculated	3	3	43,107,228	21,512,325 (49.9%)	19,343,157 (44.87%)	17,393,366 (89.92%)
BO-W3dpi_1	Susceptible	WMV-inoculated	3	1	44,760,600	22,335,730 (49.9%)	20,617,125 (46.06%)	19,031,668 (92.31%)
BO-W3dpi_2	Susceptible	WMV-inoculated	3	2	42,045,224	20,983,927 (49.91%)	18,515,499 (44.04%)	16,338,076 (88.24%)
BO-W3dpi_3	Susceptible	WMV-inoculated	3	3	43,210,794	21,561,209 (49.9%)	18,021,327 (41.71%)	15,062,225 (83.58%)
BO-0dpi_1	Susceptible	Uninoculated	0	1	44,382,722	22,152,146 (49.91%)	19,960,694 (44.97%)	17,986,581 (90.11%)
BO-0dpi_2	Susceptible	Uninoculated	0	2	43,609,702	21,761,448 (49.9%)	20,329,692 (46.62%)	18,991,998 (93.42%)
BO-0dpi_3	Susceptible	Uninoculated	0	3	40,781,540	20,347,309 (49.89%)	18,602,333 (45.61%)	17,006,252 (91.42%)

### Detection of differentially expressed genes and functional classification

**Fig. 2:**
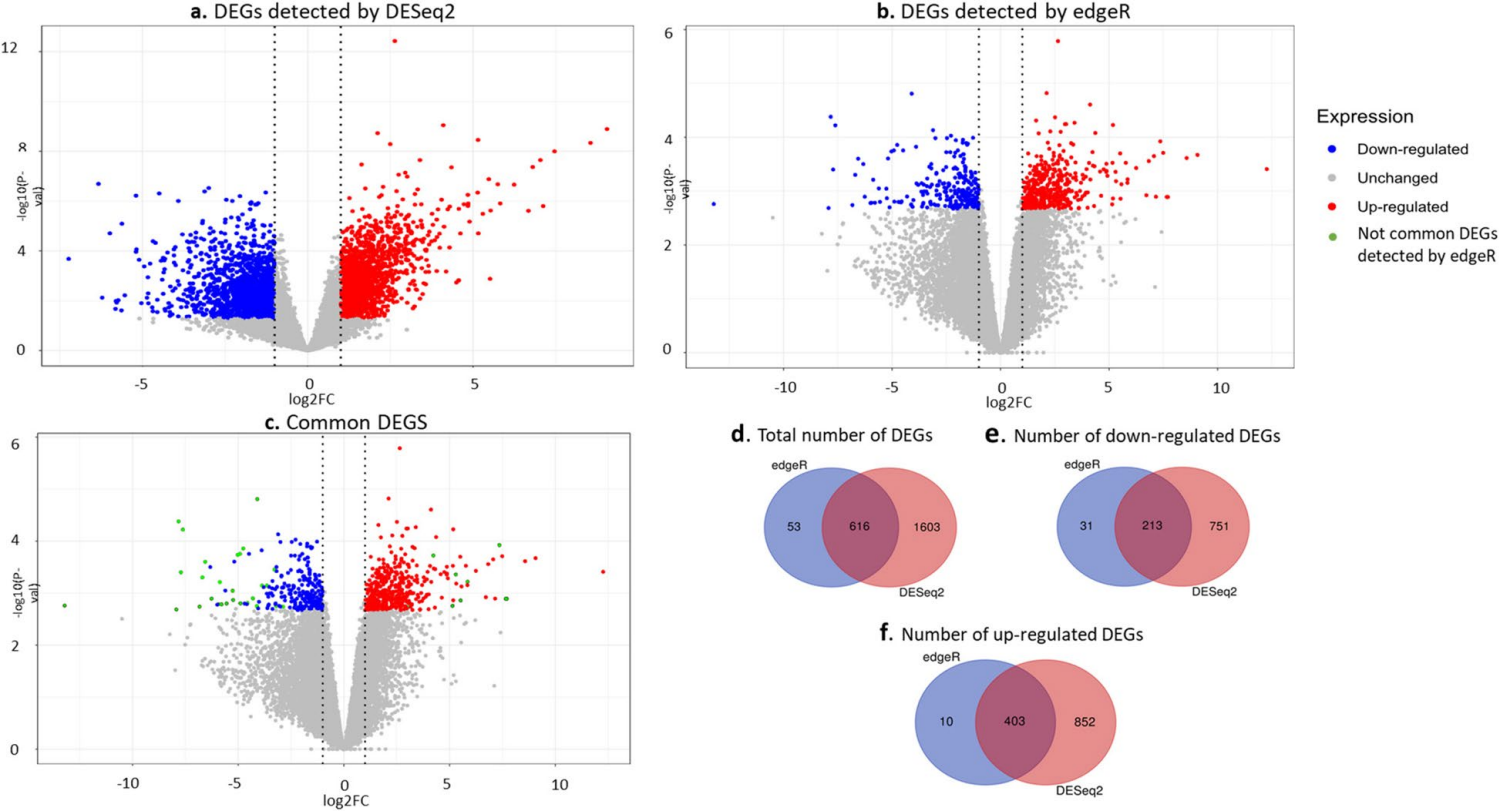
Volcano plots display differentially expressed genes (DEGs) distribution for DESeq2 (**a**), edgeR (**b**) and the consensus list between them (**c**). Red dots represent the up-regulated genes and blue dots the down-regulated. Grey dots indicate those DEGs not considered significant with *p*-value <= 0.05 and green dots show those DEGs that were not detected by both edgeR and DESeq2 methods. Venn diagrams representing the total number of DEGs detected by both edgeR and DESeq2 (**d**) and specific down- and up-regulated genes (**e**,** f**)

The 18 transcriptomes were further analyzed to detect DEGs between the resistant and susceptible genotypes when inoculated with WMV. Two different statistical methods were used (DESeq2 and edgeR) [30, 31]. In both cases, genes showing significant differences (significant p-adjusted< 0.05) for the interaction term between genotype (resistant or susceptible) and treatment (mock or WMV-inoculated) were considered as DEGs and were divided between down and up-regulated (logFC≤ − 1 and logFC≥1, respectively). DESeq2 algorithm detected 2219 DEGs (964 and 2219 down and up-regulated, respectively) (Fig. 2a**)**, while edgeR only detected 657 DEGs (244 and 413 down and up-regulated, respectively) (Fig. 2b). The consensus list of DEGs between both methods was obtained (Fig. 2c). As summary, 97.7% of the DEGs detected by edgeR (616 DEGs) were also included in the list of DEGs detected by DESeq2 (Fig. 2d). Within the down-regulated DEGs, 96.9% of those detected by edgeR were also detected by DESeq2 (213 DEGs) (Fig. 2e). The proportion was higher within the up-regulated DEGs, as a 99.2% of the DEGs detected by edgeR were also within the DEGs detected by DESeq2 (403 DEGs) (Fig. 2f). The transcriptomic dataset obtained at 0 dpi was used to check that the observed DEGs were not due to genotype differences. It was checked that none of the DEGs detected between RIL-10-3 and BO at 0 dpi was included among the list of DEGs obtained for the interaction term at 3 dpi. The consensus list of DEGs detected by both edgeR and DESeq2 at 3 dpi was used to further understand the resistance response (Additional Table 1**)**.

**Fig. 3:**
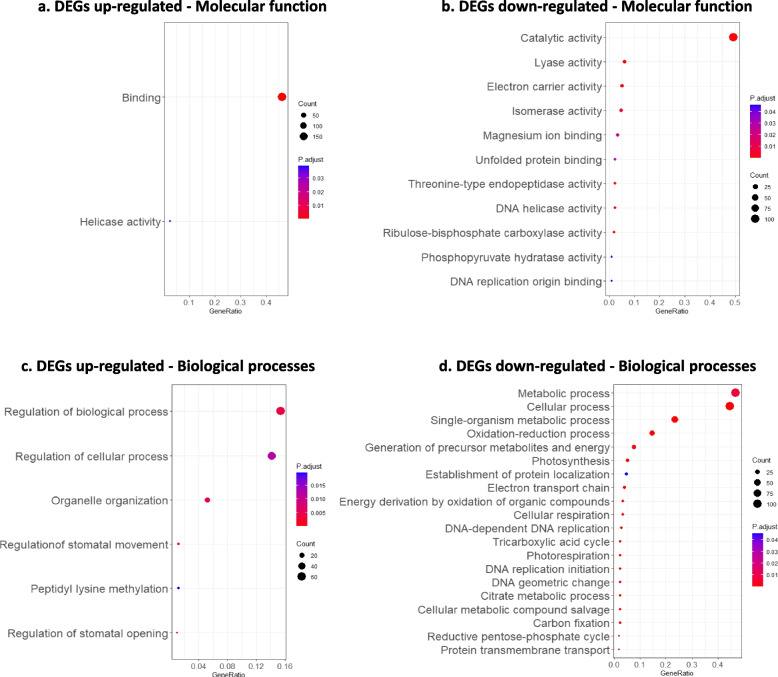
GO enrichment profile for up-(**a**,** c**) and down-represented genes (**b**, **d**). Enrichment in molecular functions (**a**, **b**), as well as, in biological processes (**c**, **d**) are indicated

DEGs were functionally annotated and GO terms enrichment analysis were performed. On the one hand, the list of up-regulated DEGs was significantly enriched (adjusted *p*-value< 0.05) in “binding” and “helicase activity” molecular functions (Fig. 3a). This list of genes was also enriched in biological processes such as “regulation of stomatal opening and movement”, “regulation of biological process”, “organelle organization” and “peptidyl-lysine methylation” (Fig. 3c). On the other hand, for the down-regulated DEGs, non-redundant functions included “ribulose-bisphosphate carboxylase activity”, “electron carrier activity”, “peptidase activity”, “lyase activity”, “catalytic activity”, “DNA helicase activity”, “isomerase activity” and “binding” (Fig. 3b). The enriched processes were mostly related to “photosynthesis”, “photorespiration”, “DNA replication” and “metabolic processes” (Fig. 3d). Even though the lists of DEGs were not enriched in “plant-pathogen interaction” or “plant defense” functions, the annotations of several DEGs were related to defense responses.

### Analysis of differentially expressed genes in the candidate regions for WMV resistance

#### Transcription changes on chromosome 11

The expression analysis was firstly focused on the region of the TGR-1551 genome associated to WMV resistance in chromosome 11, between positions 27,360,229 bp and 27,500,218 bp [18]. This QTL interval had been determined by using phenotypic data obtained in three different environments (LOD peaks values: 8.7, 11.8 and 10.8) and further fine mapped with descendance tests [18]. In this 140 kb interval, there were 12 predicted genes, some of which had annotations related to plant defense responses. Three of these genes were found to be over-expressed in the resistant genotype and none of them was down-regulated (Table 2).

**Table 2:** Predicted genes located within the candidate interval of the major QTL in chromosome 11. Log2(FoldChange) of the differentially expressed genes between the resistant and susceptible genotype for the interaction term genotype x treatment is provided

Gene name	Position (start … end bp)	Description	log2(Fold Change)
MELO3C021407	27,358,921...27,362,202	Stem-specific protein TSJT1	
MELO3C021406	27,367,916…27,369,425	basic 7S globulin-like	3.95
MELO3C031841	27,368,901…27,369,269	Unknown protein	
MELO3C021405	27,371,643…27,374,270	dual specificity protein phosphatase 1	1.82
MELO3C021404	27,393,549…27,396,064	Heavy metal-associated isoprenylated plant protein 21	
MELO3C021403	27,401,421…27,405,646	TVP38/TMEM64 family membrane protein slr0305-like	
MELO3C021400	27,428,704…27,432,334	DUF21 domain-containing protein	
MELO3C021398	27,443,667…27,449,111	serine incorporator 3	
MELO3C035181	27,444,501…27,444,779	Unknown protein	
MELO3C021397	27,451,007…27,455,813	Ribosomal protein L34e superfamily protein	
MELO3C021395	27,460,592…27,473,546	Mediator of RNA polymerase II transcription subunit 33A	1.02
MELO3C021394	27,494,941…27,501,078	Mitogen-activated protein kinase	

The highest induced expression was detected for a basic 7S globulin-like protein (MELO3C021406) with a log2FC (log2 fold change) of 3.95. A gene coding a dual specificity phosphatase 1 (MELO3C021405) was also up-regulated in the resistant RIL-10-3 with a log2FC of 1.82. The third up-regulated gene, with a log2FC of 1.02, was a mediator of RNA polymerase II transcription subunit (MELO3C021395).

Additionally, a *Vacuolar protein sorting 4* (*CmVps4,* MELO3C021413) has recently been proposed as a susceptibility factor related to the resistance against WMV derived from TGR-1551 [20]. This candidate gene is located out of the chromosome 11 candidate region proposed by Pérez-de-Castro et al. [18] and was not detected as differentially expressed in the RNA-seq.

#### Transcription changes on chromosome 5

A minor QTL with modifier effects was located on chromosome 5 (chr5: 24,607,286-27,617,536 bp) (LOD peak value: 3.3) [18]. Within this region there were 359 annotated genes. As the candidate interval of this QTL was bigger than the one of the major QTL on chromosome 11, a larger list of DEGs was obtained. There were 11 and 5 significantly up- and down-regulated DEGs in the resistant RIL-10-3, respectively (Table 3) (Suppplementary Table 1). Among the up-regulated DEGs there were 7 of them whose annotations had been related to plant defense functions: an importin subunit alpha (MELO3C004204), a 5–3 exoribonuclease (MELO3C004356), a prenylyltransferase superfamily protein (MELO3C004366), a calcium uptake protein (MELO3C004433), a serine-rich protein-like protein (MELO3C004434), a transmembrane protein (MELO3C004435) and a ubiquitin family protein (MELO3C004438). On the other hand, the annotation of all the down-regulated genes in this region had been associated to responses to biotic stresses: a calreticulin protein (MELO3C004194), a GTP-binding protein SAR1A (MELO3C004196), a terpene cyclase/mutase family member (MELO3C004329), a thioredoxin-like protein (MELO3C004371) and a DNA helicase (MELO3C004448).

**Table 3:** Predicted genes located within the candidate interval of the major QTL in chromosome 5 that have been detected as differentially expressed. Log2(FoldChange) of the differentially expressed genes between the resistant and susceptible genotype for the interaction term genotype x treatment is provided

Gene name	Position (start … end bp)	Description	log2(Fold Change)
MELO3C004194	24,886,205…24,890,478	calreticulin	−1.64
MELO3C004196	24,898,167…24,901,559	GTP-binding protein SAR1A	−1.52
MELO3C004200	24,947,277…24,952,407	E3 SUMO-protein ligase NSE2	1.48
MELO3C004204	24,983,611…24,989,254	Importin subunit alpha	2.15
MELO3C004219	25,131,665…25,134,287	Dormancy/auxin associated protein	3.16
MELO3C004305	26,042,063…26,048,099	Pre-mRNA-splicing factor SLU7	2.97
MELO3C004329	26,298,777…26,308,596	Terpene cyclase/mutase family member	−2.59
MELO3C004356	26,610,676…26,621,447	“5–3 exoribonuclease”	1.14
MELO3C004366	26,689,354…26,697,690	Prenylyltransferase superfamily protein	2.15
MELO3C004371	26,731,110…26,735,263	Thioredoxin-like protein 1	−1.24
MELO3C004421	27,112,362…27,117,173	L-allo-threonine aldolase	4.36
MELO3C004433	27,203,527…27,207,131	calcium uptake protein 1, mitochondrial-like isoform X1	5.18
MELO3C004434	27,208,137…27,211,375	Serine-rich protein-like protein	4.12
MELO3C004435	27,217,111…27,221,085	Transmembrane protein	1.88
MELO3C004438	27,252,281…27,262,164	Ubiquitin family protein	2.30
MELO3C004448	27,326,640…27,332,555	DNA helicase	−4.49

### Expression of known genes related to plant defense responses

The gene responsible of WMV resistance in *Arabidopsis thaliana* has been cloned and it encodes a nucleus-encoded chloroplast phosphoglycerate kinase [32]. Its ortholog gene in melon (MELO3C019634) was not deregulated and neither were genes with the same annotated function (Additional Table 1). Same happened with the genes related to WMV resistance in cucumber. In this crop, a QTL linked to the recessive gene *wmv*^*02245*^ was mapped on chromosome 6 [33]. The 134.7 kb physical distance of this region included 21 candidate genes, 16 of which were annotated. Five of those candidate genes were related to plant defense functions and included 2 zinc finger structures, 2 nucleic acid and protein binding sites and a pathogenesis-related transcriptional factor. The ortholog genes in melon were mainly located on chromosome 5 but they were located outside the candidate region derived from TGR-1551 and they were not deregulated.

**Fig. 4:**
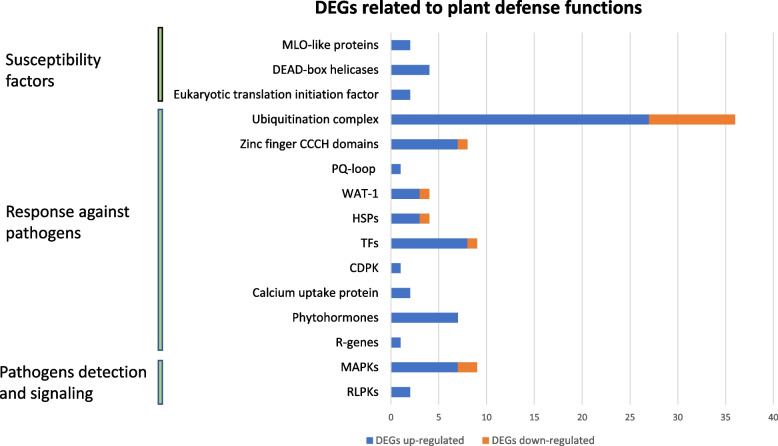
Number of differentially expressed genes (DEGs) up- and down-regulated whose annotation has previously been related to different plant defense functions

To further identify candidate genes related to WMV resistance, the expression profile of genes associated with plant defense responses against viruses were studied. We looked for R-genes previously characterized in melon [34], families of transcription factors (TFs) involved in stress responses, pathogen-resistant proteins, genes involved in gene silencing and hormonal signaling and susceptibility factors (Fig. 4).

Among the 70 characterized R-genes in *C. melo* [34], none of them were deregulated. Neither were an additional set of selected genes conferring resistance to pathogens [25].These resistance genes were located out of the boundaries of the candidate QTLs. Moreover, in other resistance studies carried out in cucurbits, these resistance genes were mostly deregulated at latter infection stages (6 and 12 dpi) [24, 25]. On the other hand, protein kinases are known to mediate the signaling mechanisms required for the defense response, including the activation of TFs and systemic responses. Among the up-regulated DEGs there was one located in chromosome 5 coding a disease resistance protein (MELO3C008572) (Additional Table 1), seven genes coding mitogen-activated (MAPKs) or serine/threonine protein kinases (MELO3C021470, MELO3C012233, MELO3C013739, MELO3C010334, MELO3C019687, MELO3C013322 and MELO3C026640), as well as two receptor protein-kinases (RPKs) (MELO3C002351 and MELO3C007457) and a calcium-dependent protein kinase (CDPK) (MELO3C017756) (Fig. 4). Many other kinases were also overexpressed (Additional Table 1). Among the down-regulated DEGs, there were two genes coding a mitogen-activated or serine/threonine-protein kinase (MELO3C026848 and MELO3C003047) and a dual specificity phosphatase (MELO3C024481).

There were other DEGs whose functional annotation had also been described as related to plant defense responses. Among these genes there were heat-shock proteins (HSP) (MELO3C007297, MELO3C016712, MELO3C021100 and MELO3C008865), which are molecular chaperones that protect plants from damage to diverse stresses; WAT1-related proteins (MELO3C010177, MELO3C010471, MELO3C009934 and MELO3C012015) that have been associated to resistance against the *cucumovirus* cucumber mosaic virus (CMV) in pepper [35]; or protein domains that form part of some atypical R proteins, such as PQ-loop repeat proteins (MELO3C020942) or zinc finger CCCH domains [36–39] (Fig. 4; Additional Table 1**)**.

#### Transcription factors

Among the 58 transcription factors (TFs) families described in plants, six major TFs families have been reported as involved in stress responses [40]. We looked for DEGs coding TFs implied in plant-defense functions and two ERFs (ethylene responsive transcription factors) (MELO3C007572 and MELO3C021306), two bHLH (basic helix-loop-helix) (MELO3C023299 and MELO3C005178), two bZIP (basic leucine zipper) (MELO3C012961 and MELO3C015377), one MYB (myeloblastosis related) (MELO3C024440) and one NAC (no apical meristem (NAM)) (MELO3C012391) were up-regulated, while only one MYB was down-regulated (MELO3C012039). Thus, a strong de-regulation of the TFs expression profile was confirmed in the resistant genotype even at early infection stages.

#### Hormones

Phytohormones, including ethylene (ET), salicylic acid (SA), jasmonate (JA), abscisic acid (ABA), brassinosteroids (BR), cytokinin (CK) or auxins (AUX) play essential roles by activating the plant defense response against viruses. We looked for DEGs coding proteins related to phytohormone response or synthesis. In addition to the previously named TFs affected by JA or ET accumulation (see previous section), two genes coding stem-specific protein TSJT1 (MELO3C007297 and MELO3C016712) were up-regulated. These proteins participate in the systemic acquired resistance (SAR) through the SA mediated signaling pathway [41]. There was also an over-expressed gene coding a glycosyltransferase (MELO3C009339), which could be involved in the SA cycle by modulating N-hydroxy-pipecolic acid (NHP), a regulator for plant innate immunity and SAR [42]. Finally, four genes coding proteins related to AUX were also over-expressed (Additional Table 1) (Fig. 4**).**

#### Ubiquitination and ubiquitin/proteasome system complex

The ubiquitin proteasome system (UPS) constitutes an important part of plant responses to viruses. It can target different viral components to prevent virus spread, inhibit viral replication or to mitigate disease symptoms [43, 44]. There were 27 over-expressed genes encoding proteins that are part of the UPS. Among the coded proteins there were 4 ubiquitin-conjugation enzymes, 11 ubiquitin-protein ligases (including RING-type) and 9 F-box proteins. RING-type ligases and F-Box proteins are UPS key factors, since they, respectively, define the substrates for ubiquitination and induce the hypersensitive resistance response [45]. There were 7 and 2 additional DEGs that were down-regulated and coded different proteasome subunits and F-box proteins, respectively (Fig. 4) (Additional Table 1).

#### RNA silencing

RNA silencing constitutes an important defense method against viral infections. Even though potyvirus code a HC-Pro silencing suppressor that puts down the miRNA pathway [46], it has been proposed that silencing may play a key role in the resistance response of TGR-1551 to WMV [22]. We searched for DEGs involved in this mechanism but there were not DEGs coding proteins directly implied in the silencing machinery, such as DICER-like genes, ARGONAUTE genes or genes involved in the RNA-induced silencing complexes.

#### Susceptibility factors

Cellular translation factors are recruited by plant viruses to both translate their viral RNAs and to control their replication and movement through the plant. Hence, mutations in these proteins can lead to broad spectrum resistances [47, 48]. Additionally, other translation factors such as eIF4A-like helicases (a DEAD-box ATP- dependent RNA helicase) are also frequently used by viruses [49] and can act as effectors by blocking RNA virus replication [50]. Genes coding one eukaryotic translation initiation factor-like protein (MELO3C002515), one translation initiation factor 4E (MELO3C026612) and four DEAD-box ATP-dependent RNA helicases (MELO3C023052, MELO3C009973, MELO3C017907, MELO3C006599) were over-expressed in the resistant RIL 10–3 compared to the susceptible cultivar BO (Fig. 4**)** (Additional Table 1). There were not translation factors downregulated.

Moreover, *CmVps4* (MELO3C021413) and *CmVps41* (MELO3C004827) had been proposed as a susceptibility factors conferring resistance against WMV and CMV systemic infections in melon [20, 51, 52]. Additionally, a VPS4-like gene has also been proposed as a candidate gene conferring resistance to zucchini yellow mosaic virus (ZYMV) in cucumber [53]. Those genes were not detected as DEGs in the RNA-seq study. Giner et al. [51] previously indicated that *CmVps41* expression was not different between susceptible and resistant genotypes upon infection. Two genes annotated as vacuolar protein sorting-associated proteins (MELO3C005953 and MELO3C032233) were up-regulated in the RIL-10-3 (Additional Table 1).

### qRT-PCR validation of DEGs

**Fig. 5:**
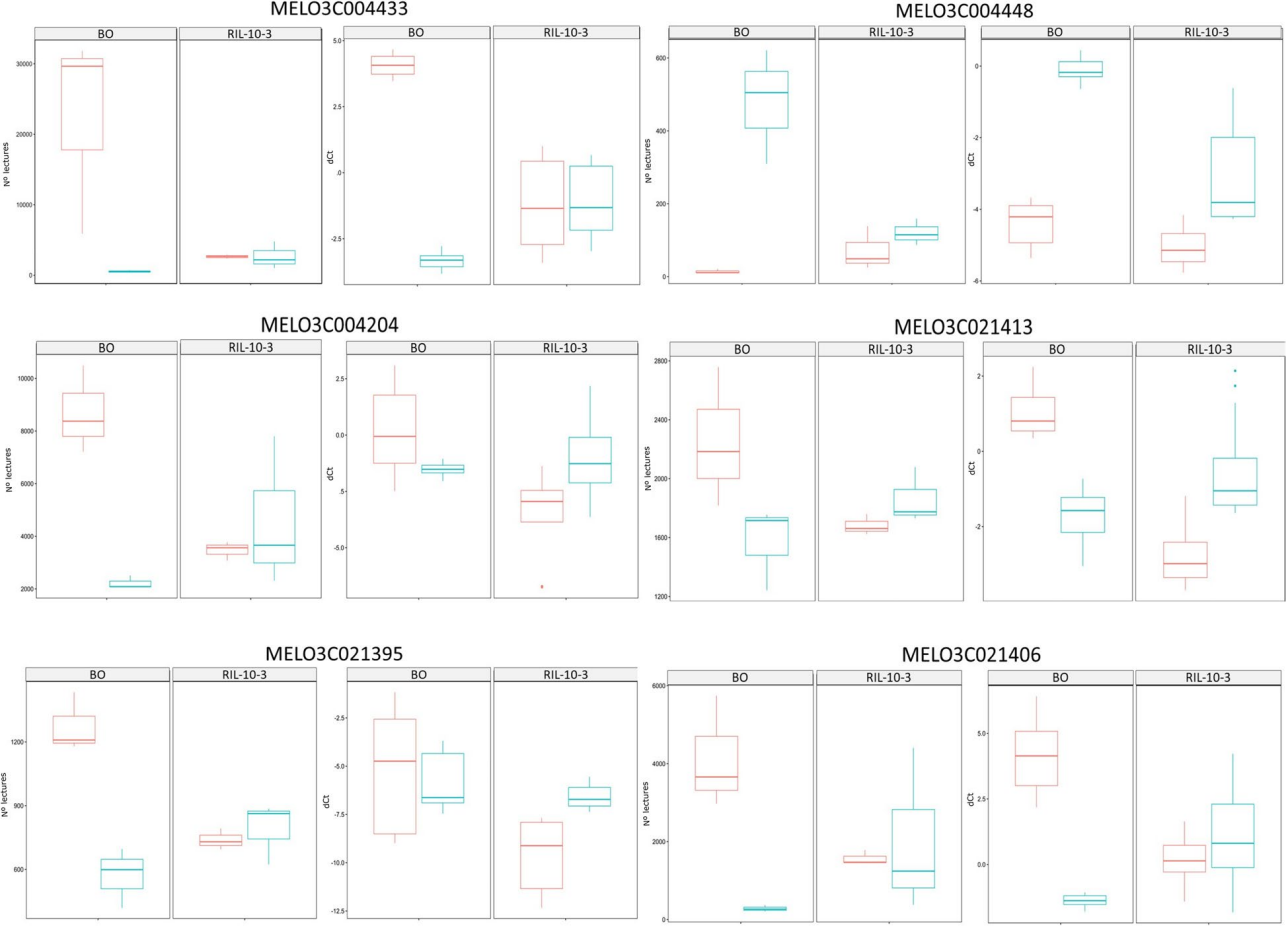
Comparation of the expression profiles of 6 candidate genes for WMV resistance obtained by both RNA-seq and RT-qPCR. Left panels of each gene represent the number of lectures detected by RNA-seq, while right panels indicate their relative expression (dCt) determined by RT-qPCR. The central line within the box represents the median expression value. The box encompasses the interquartile range (IQR). The whiskers extending from the box indicate the minimum and maximum values within 1.5 times the IQR. Data points beyond the whiskers are represented as individual points and are considered outliers

In total, 6 significant differentially expressed genes were selected for qRT-PCR validation (Fig. 5). The selected genes were located on chromosomes 11 (MELO3C021395, MELO3C021406 and MELO3C021413) and 5 (MELO3C004433, MELO3C004448, MELO3C004204). The normalized relative accumulation of their transcripts measured by RT-qPCR was compared to the number of lectures detected by RNA-seq, showing similar expression patterns relative to the genotype x treatment term. The ANOVA tests showed that there were significative differences for the genotype x treatment interaction term for the 6 studied genes (p.value < 0.05). This is consistent with the results obtained for the RNA-seq analysis of all the genes (Table 2 and Table 3) except for MELO3C021413, which was not detected as differentially expressed in the transcriptomic assay. However, when the expression patterns of both RNA-seq and RT-qPCR data were compared a similar expression trend was observed. The differential expression analysis conducted with the RNA-seq data considers the expression profiles of all the genes in the genome to determine if each one of them is differentially expressed. This can lead to underestimate the differences, which explains the significance differences between both methods when a similar expression trend is observed. The RT-qPCR results confirmed the high reproducibility of the obtained transcriptomic data.

### SNPs linked to DEGs associated with WMV response

**Fig. 6:**
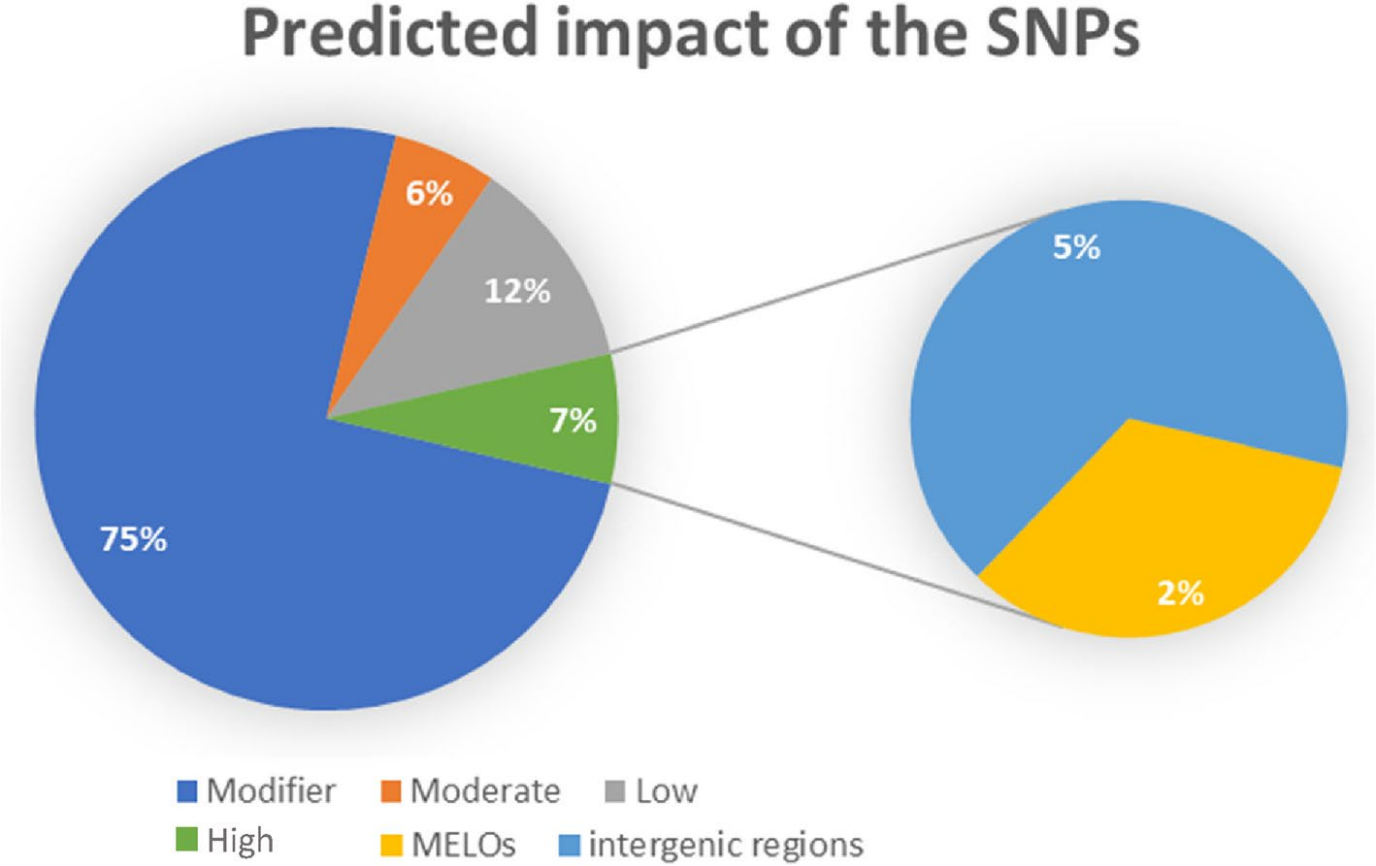
Representation of the percentage of detected SNPs with different predicted impacts on the protein function

Out of the 198,881 variants detected between the RIL-10-3 and BO samples, multiallelic SNPs were discarded, so only 192,561 were considered. There were 303,694 (75.26%), 23,171 (5.74%), 47,764 (11.84%) and 28,874 (7.16%) variants with a modifier, moderate, low and high predicted impact, respectively (Fig. 6). Of the 28,874 variants with a predicted high impact effect, 9708 (33.63%) were associated to gene coding sequences. Moreover, SNPs were detected in 401 of the 403 (99.5%) DEGs that were up-regulated, and 334 of those variants were associated with a predicted high impact effect (i.e., appearance of codon stops, open reading frame shifts, amino acid changes not favored by evolution…). Regarding to the 213 DEGs that were down-regulated, SNPs with a high impact were detected in 152 of them (71,36%).

SNPs variants with a high predicted impact were observed in the three DEGs detected in the candidate region of chromosome 11 (MELO3C021405, MELO3C021406 and MELO3C021395) (Table 4**) (**Additional Table 2). Among others, those effects were due to the gain of a premature start codon gain, the appearance of a missense variant causing an aminoacidic change and modifications of the splicing regions. The gene MELO3C021395 (*MED33A*) was the one in which more high-impact variants were detected **(**Table 3). Additionally, SNPs were detected in the coding regions of all the genes located within the candidate interval. As for the *vacuolar protein sorting 4* (MELO3C021413), proposed as a susceptibility factor, 6 high impact variants were observed (Additional Table 2). Nevertheless, it had been proposed that a single non-synonymous substitution in *CmVps4*^*P30R*^ conferred the resistance to WMV [20]. This mutation is caused by a [T/C] SNP in the genomic position Chr11:27,319,260 bp (v.4.0) but both BO and the RIL-10-3 have a cytosine in this position. These results were also confirmed by sequencing by Sanger this genomic region of BO, TGR-1551 and RIL-10-3, obtaining the same result. The presence of a cytosine in this genomic position is translated into a proline (P), instead of into an arginine (R). Thereby, the previously described change in TGR-1551 *CmVps4*^*P30R*^ was not present in our resistant accession. However, any of the other high impact SNPs detected at within this gen sequence could also be responsible of the resistance.

**Table 4:** Summary of the SNPs observed within the sequences of the genes located in the candidate region of chromosome 11 and among the DEGs detected within the QTL in chromosome 5. The predicted impact and the effect of the SNPs is indicated

Gene name	Chr	log2(Fold Change)	Variants impact				Variants effect						
			High	Low	Moderate	Modifier	Premature start codon gain	Premature stop codon gain	Frameshit variant	Missense variant	Splice acceptor variant	Splice donor variant	Splice region variant
MELO3C004194	5	−1.64	3	3	2	12	0	0	0	1	0	3	6
MELO3C004196	5	−1.52	2	8	1	20	0	0	1	1	0	1	7
MELO3C004200	5	1.48	2	5	5	8	0	0	0	5	0	2	2
MELO3C004204	5	2.15	8	15	2	21	1	1	1	2	2	5	15
MELO3C004219	5	3.16	3	2	1	22	1	1	0	1	1	1	3
MELO3C004305	5	2.97	5	3	0	27	0	0	0	0	1	4	6
MELO3C004329	5	−2.59	0	0	2	33	0	0	0	2	0	0	0
MELO3C004356	5	1.14	0	3	2	30	1	0	0	2	0	0	1
MELO3C004366	5	2.15	3	3	0	29	0	0	0	0	1	2	4
MELO3C004371	5	−1.24	1	0	0	37	0	0	1	1	1	0	1
MELO3C004421	5	4.36	4	6	4	32	0	0	0	4	1	3	8
MELO3C004433	5	5.18	7	9	7	102	1	1	3	9	2	2	6
MELO3C004434	5	4.12	0	0	1	101	0	0	0	1	0	0	0
MELO3C004435	5	1.88	12	8	7	17	1	0	2	9	4	7	12
MELO3C004438	5	2.30	10	16	2	11	0	1	1	3	5	5	21
MELO3C004448	5	−4.49	1	1	0	13	0	0	0	0	0	1	1
MELO3C021407	11		2	7	1	10	0	0	1	2	0	1	6
MELO3C021405	11	1.82	1	2	1	27	1	0	0	1	0	1	1
MELO3C021406	11	3.95	1	2	8	15	0	0	1	7	0	0	0
MELO3C031841	11		0	0	3	23	0	0	0	3	0	0	0
MELO3C021404	11		3	9	12	10	0	0	2	7	1	0	2
MELO3C021403	11		0	2	1	5	0	0	0	1	0	0	1
MELO3C021400	11		2	5	1	19	0	0	0	1	0	2	5
MELO3C035181	11		0	0	1	15	0	0	0	1	0	0	0
MELO3C021398	11		1	5	2	16	0	0	0	2	0	1	3
MELO3C021397	11		0	0	2	37	0	0	0	2	0	0	0
MELO3C021395	11	1.02	9	12	10	58	0	0	2	11	3	4	8
MELO3C021394	11		8	15	0	26	0	0	0	0	2	6	18

Regarding the DEGs identified within the QTL candidate region in chromosome 5, SNPs with a high predicted impact were detected in all of them except for the genes MELO3C004329, MELO3C004356 and MELO3C004434 (Additional Table 2).

Moreover, at least one SNP with a predicted high effect hit all the previously mentioned DEGs except for the down-regulated MYB transcription factor (MELO3C012039), that was affected by variants with low, moderate and modifier effects. These changes in the genomic sequences could affect the expression of those genes and the activity of the coded proteins.

### Weighted gene co-expression network analysis

Weighted gene co-expression network analysis (WGCNA) produced 19 gene clusters (GCs) (Additional Fig. 1). GC6 cluster was the only one showing a genotype-specific pattern of gene co-expression. Moreover, there were seven clusters (GCs 1, 9, 10, 13, 14, 17 and 19) that showed statistically significant differences regarding the interaction term between genotype and treatment. Most up-regulated genes in the RIL-10-3 within these GCs were enriched in KEGG pathways associated to plant hormone signaling transduction, MAPK signaling pathway, spliceosome, ubiquitination, and basal transcription factors, whereas the genes that had an expression profile negatively correlated with the clusters were mainly classified in ontologies related with replication, photosynthesis, carbon metabolism, including the TCA cycle, and ribosome (Additional Fig. 2).

## Discussion

WMV is one of the most limiting factors for melon production worldwide, as it affects all the main producing areas. Moreover, this potyvirus is constantly evolving, and new and more virulent strains continue to appear [3, 5, 54]. The African accession TGR-1551 is the most promising resistance source against WMV. In works developed by our research group, a major QTL related to the recessive resistance was mapped to a 140 kb region, containing 12 predicted genes, in chromosome 11. Additionally, a minor QTL with modifying effects was also mapped to a wider region in chromosome 5 [18]. Previous microarray studies revealed a huge transcriptomic remodeling related to this resistance [21] and a RNAome assay highlighted the possibility that silencing mechanisms could also be implied in the immune response [22]. In this study, a resistant and a susceptible genotype sharing approximately a 50% of their genome were mock- and virus-inoculated, and we took advantage of the assets offered by RNA-seq compared to microarrays, to take a closer look at the transcriptional changes after WMV infection, trying to correlate the early changes in gene expression to the resistance response.

When we compared the genotype x treatment interaction term, 616 common DEGs were obtained with two different algorithms, edgeR and DESeq2. Out of those 616 genes, 403 and 213 were up- and down-regulated, respectively. This is consistent with the huge transcriptomic remodeling previously observed after inoculation of resistant genotypes with WMV [21], ZYMV [27] and tomato leaf curl New Delhi virus (ToLCNDV) [25], respectively. Moreover, 3 and 16 DEGs were identified within the major and minor QTL regions in chromosomes 11 and 5, respectively.

In the chromosome 11 region, the 3 DEGs were all up-regulated in the resistant RIL-10-3. Among them, the gene with the highest fold change difference presented a frameshift mutation in the RIL-10-3, and coded a basic 7S globulin-like protein (Bg7S; MELO3C021406). Even though Bg7S were initially thought to only be seed storage proteins, they have been proven to be multifunctional [55]. In tomato, Bg7S inhibits xyloglucan-specific endo-β-1,4-glucanase (XEG), a cell wall-degrading glucosyl hydrolase derived from *Aspergillus aculeatus* [56]. In soybean, Bg7S is expressed in response to biotic and abiotic stressors and it has been shown to have protein kinase activity [57]. However, despite the multiple effects described for this protein, to our knowledge, Bg7S have not previously been related to resistance responses against viruses.

Another DEG located within the candidate region in chromosome 11 coded a dual specificity phosphatase 1 (MELO3C021395), and 9 variants with a predicted high impact were found within its sequence. A gene with the same predicted function was also found in the WMV resistance candidate region in cucumber [33]. Dual specificity phosphatases are a sub-class of MAPK phosphatases (MKPs) whose main function is to ensure an appropriate balance stress signaling and suppression of autoimmune-like responses by negatively modulating the MAPK kinetics [58–62]. They are involved in controlling plant growth and development as well as modulating stress adaptation [61]. Some dual-phosphatases also modulate phytohormone signal transduction pathways, especially those related to auxins, SA and ABA [59, 63, 64]. Nevertheless, these proteins have always been associated with higher resistance levels when their expression is repressed or by avoiding autoimmune damages. Contrary to what was expected, in this assay this gene was over expressed in the resistant genotype, and no necrotic damages associated with the immune response have been reported in the susceptible cultivars.

The third gene that was up-regulated in the candidate region of chromosome 11 coded a mediator of RNA polymerase II transcription subunit 33A (*MED33A*; MELO3C021395). A premature start codon gain was detected within its coding sequence. Mediator is a large multi-subunit complex that integrates input signals from different pathways and connects them to the RNA polymerase II (RNAPII). Mediator complex plays a key role in fine-tune pathway- and gene-specific transcriptional reprogramming by acting as a hub between TFs and RNAPII [65]. *MED33A* (REF4-related 1; RFR1) and *MED33B* (reduced epidermal fluorescence 4; REF4) subunits are implied the regulation of the phenylpropanoid pathway (PPP), acting as repressors [66, 67]. Knock-out mutants of the *MED33A* and *MED33B* subunits showed an increased expression of genes such as phenylalanine-ammonia lyase 1 (PAL1), PAL2, cinnamate 4-hydroxylase (C4H) and 4CL1, that are implied in the early phenylpropanoid biosynthetic pathway [68]. PPP-derived metabolites play diverse roles in plant defense and are often positively correlated with resistance. In fact, downstream of the core PPP, accumulation of PPP-derived phytoalexins are common resistance mechanisms [69–74]. Nevertheless, the perturbation of the PPP through the application of the C4H inhibitor piperonylic acid (PA) in tomato triggered systemic, broad-spectrum resistance by systemically inducing immune signaling and pathogenesis-related genes and locally activating the production of reactive oxygen species (ROS) [75]. Thus, the over-expression of *MED33A* in the resistant RIL-10-3 and the consequent repression of the PP synthesis could lead to an early strong defense response to WMV.

Moreover, several mediator subunits are directly related to plant defense functions by both relaying signals from upstream regulators and by transmitting phytohormone signals [76, 77]. Some mediator subunits such as *MED18* and *MED25* have been directly related to virus defense in *A. thaliana*. Both subunits are implied in the JA signaling pathway but their silencing affects virus infection differently. Whereas *MED18* is considered a susceptibility factor, *MED25* is required for defense against virus infection. The up-regulation of *MED33A* in the resistant genotype could indicate that this subunit would also be implied in an active defense response against WMV. Recently, a *Vacuolar sorting 4* (*CmVps4*) (MELO3C021413) has been proposed as a susceptibility factor to WMV in melon [20]. This gene was located out of the chromosome 11 candidate region proposed by Pérez-de-Castro et al. [18]. It was not deregulated in the RNA-seq assay, and the RT-qPCR data showed that it was slightly up-regulated in the resistant RIL-10-3 which would be contrary to the expression patterns showed by other susceptibility factors, where this kind of genes are over-expressed in the susceptible genotypes under infection conditions [78, 79]. However, studies conducted with other vacuolar sorting proteins described as susceptibility factors showed that there was no difference in gene expression between susceptible and resistant genotypes during infection [51]. We also observed that both TGR-1551 and RIL-10-3 genotypes did not have the mutation *CmVps4*^*P30S*^ that is supposed to confer the resistance. Instead TGR-1551, RIL-10-3 and BO carried the *Vps4*^*Wt*^ allele, which has also been observed in the resistant accession PI 414723 but that was not related to the resistance derived from TGR-1551. However, any of the other high impact SNPs detected within the coding region of this gen could also be responsible of the resistance. These discrepancies between the TGR-1551 genotypes could be explained by the fact that this wild relative was found in open-pollinated populations, were the level of heterogenicity is higher and different processes of self-pollination have resulted in these differences. Either way, *CmVps4* should not be discarded as a candidate gene involved in WMV resistance. However, considering the great transcriptomic remodeling observed in this work, additional resistance mechanisms could be implied in the defense response.

Within the chromosome 5 candidate region there were also several DEGs whose annotated functions had previously been associated to resistance responses against pathogens. Ubiquitin family proteins (MELO3C004438) can target different viral components to prevent virus spread, inhibit viral replication or to mitigate disease symptoms [80, 81]. Transmembrane proteins (MELO3C004435) and importins (MELO3C004204) can act as susceptibility factors and have also been related to silencing responses [82–85]. Exoribonucleases (MELO3C004356) can negatively regulate the accumulation of viruses [86, 87]. Calreticulins (MELO3C004194) are a kind of chaperones that binds to calcium and have been associated to the defense response against biotrophic pathogens [88, 89]. They are essential to the correct maturation of some surface glycosylated receptors. The gene coding a terpeno cyclase/mutase family member (MELO3C004329) was down-regulated in our resistant genotype, but these kind of genes have been found to be over expressed in the resistant accession WM7 when it was inoculated with ToLCNDV [25]. As it happened with *MED33A*, these proteins are implied in the PPP [90]. A gene coding a thioredoxin-like protein (Trxs) (MELO3C004371) was also down-regulated in the RIL-10-3. Trxs can contribute to plant defense by expressing defense responsive pathogenesis-related (PR) genes [91] but have also been described as negative regulators of ROS production [92, 93]. Finally, calcium uptake proteins (MELO3C004433), GTP-binding proteins (MELO3C004196) and serine-rich proteins (MELO3C004434) have been related to the transduction of signals after the recognition of pathogen associated molecular patterns (PAMPs) [94–98]. Moreover, calcium signals also play an important role in the second layer of defense called effector-triggered immunity (ETI) [99]. It has been observed that the calcium signal can be downstream translated into outputs such as gene expression or stomatal closure [100]. In this sense, it is worth saying that the regulation of stomatal movement and opening were two of the enriched biological processes among the DEGs that were up-regulated. The expression pattern differences between the resistant and susceptible genotypes might be due to the accumulation of SNPs with a predicted high impact in the coding regions of all the cited DEGs. Moreover, the minor QTL on chromosome 5 is located within a resistance cluster [18, 101]. This could explain the huge transcriptomic remodeling observed in this region in the resistant genotype after WMV-infection, as several genes could work in a synergistic manner to improve the resistance offered by the major gene on chromosome 11.

In addition to the changes observed within the two candidate regions, a huge transcriptomic remodeling was also observed across the whole genome, which is consistent with previous microarray data [21]. Even though the gene ontologies of the detected DEGs were not enriched in plant defense related functions, numerous genes previously classified as susceptibility factors, or related to the pathogen’s detection and transduction of signals and the response against viruses were differentially expressed. This is consistent with the fact that several gene clusters detected with WGCNA were enriched in molecular functions such as “plant-pathogen interaction”, “plant hormone signal transduction”, “MAPK signaling pathway”, “basal transcription factors” or “ubiquitin mediated proteolysis”. The up-regulation of these biological processes had also been observed in other cucurbits infected with viruses [23, 25–27]. Moreover, both the list of DEGs and some GCs that were down-regulated in the resistant genotype were enriched in gene ontologies related to the photosynthesis and the basal metabolism. The activation of defense responses is energetically expensive, which is why it is often done to the detriment of photosynthesis and the assimilatory metabolism, specially at early infection stages [102]. In summary, these results showed that an active fight against WMV is taking place in the resistant RIL-10-3.

## Conclusion

This work presents the first RNA-seq study of the transcriptomic changes due to WMV infection in cucurbits, concretely in melon. In total, 616 genes were detected as differentially expressed between the resistant and the susceptible genotype. This analysis has allowed the identification of the gene MELO3C021395, which coded a mediator of RNA polymerase II transcription subunit 33A (*MED33A*), as a candidate gene conferring resistance against WMV. Moreover, the WGCNA performed on the global gene expression dataset detected 19 GCs, of which 7 were differentially expressed. The inoculation of WMV triggered a huge transcriptomic remodeling in the resistant genotype, including genes located within and out of the previously described resistant QTLs. The early response turned out to be comprehensive, including genes involved in plant-pathogen interaction, plant hormone signal transduction, the MAPK signaling pathway or ubiquitin mediated proteolysis. As a consequence of the activation of these mechanisms, the photosynthetic pathway, as well as the synthesis of basal metabolites, was altered in the resistant genotype. These results will be useful to better understand the mechanisms underlaying the resistance response against WMV in melon.

## Methods

### Biological materials

The WMV resistant RIL 10–3, derived from the initial cross between the resistant accession TGR-1551 (*C. melo*, acidulus group) and the susceptible Spanish cultivar “Bola de oro” (BO) (*C. melo*, ibericus group), and its parental line BO were used as plant materials in this study. This resistant RIL-10-3 carried the regions on chromosomes 11 and 5 previously linked to the resistance to WMV. Moreover, RIL-10-3 and BO shared approximately a 50% of their genome, which is useful to reduce the background noise in an RNA-seq assay. Seeds of both lines were germinated following the protocol described in [25] to ensure a homogeneous germination. The plants were grown in a growth chamber under controlled conditions of 27 °C, 16 h/8 h of light and darkness, respectively, and watering as needed.

The WMV virus used in this assay was originally collected in melon infected plants in Huerta de Vera fields (Valencia, Spain). This isolate, WMV-Vera, has been characterized and it belongs to the “emerging” group [103] (GenBank: MH469650.1).

### Sampling design, inoculation and symptoms assessment

At the two true-leaves stage, 24 seedlings of both susceptible and resistant genotypes were mechanically inoculated with isolate WMV-Vera. The inoculum was prepared by crushing symptomatic leaves of infected melon plants. Inoculation was performed by rubbing the leaves with a swab with the inoculum, an inoculation buffer and carborundum as described by [104]. The same number of plants were mock-inoculated following the same protocol but using only the inoculation buffer and carborundum. Plants were cultivated for 30 days after mechanical inoculation (dpi) under controlled conditions previously described. Four different plants per treatment (susceptible/resistant genotype x virus/mock inoculated treatment) were sampled at 0, 3, 7, 10 and 15 days after inoculation (dpi). For each plant, a leaf disc was collected in a 1.5 ml microtube tube from each of the expanded leaves but the inoculated leaves. Samples were immediately frozen in liquid nitrogen and stored at − 80 °C. All the plants were maintained until 30 dpi to phenotype the symptoms.

Additionally, at 0 dpi (two true-leaves stage) (i.e., before inoculation), all expanded leaves of three healthy seedlings of each genotype were collected and used as the control treatment, maintaining those plants alive with their apex intact. Sampling was performed as previously described.

Symptoms of WMV infection were assessed at every sampling point (3, 7, 10 and 15 dpi), with a scale from 0 (no symptoms) to 4 (severe mosaic and leaf distortion). Virus infection also was assessed by RT-qPCR, following the method described in section 5.5 (Validation of differentially expressed genes by RT-qPCR), and using the primers WMV-CE-170: TGTTGCTTCATGGAAGATTGGT and WMV-CE-171: AAAATTGTGCCATCAGGTGCTA.

### RNA extraction and library preparation

For the transcriptomic analysis, three biological replicates at 0 and 3 dpi were selected (i.e. 18 samples = 3 replicates × 2 genotypes at 0 pdi + 3 replicates × 2 genotypes × 2 treatments at 3 dpi). In all 3dpi samples presence or absence of WMV was confirmed by RT-qPCR.

Total RNA was extracted using 700 μL of Extrazol® EM30 (Blirt DNA, Gdansk, Poland) according to kit’s specifications. RNA integrity was checked by 1.5% agarose gel electrophoresis, and purity and quantity were measured using a NanoDrop 1000 (Thermo Scientific, Waltham, MA, USA). Total RNA (2 μg) of the selected 18 plants was sent to Macrogen Inc. (Seoul, Republic of Korea) for cDNA library construction. RNA integrity was measured using an Agilent Technologies 2100 Bioanalyzer with an RNA Integrity Number (RIN) value ≥7 and an RNA ratio ≥ 1. Approximately 1 μl of total RNA was used to construct the RNA-seq libraries using the TruSeq Stranded mRNA LT Sample Prep Kit (Illumina) by following manufacturer’s instructions. Finally, libraries were sequenced (paired-end 150 bp) using a NovaSeq6000 Sequencing System (Illumina, CA, USA) and producing more than 40 million reads per sample.

### RNA-seq analysis and differentially expressed genes analysis

Quality of raw sequences was checked using FastQC v0.11.9 [105]. Sequences were processed using Trimmomatic 0.38.0 [106], to remove adapters and low-quality reads. Quality of the trimmed and clean reads was checked again with FASTQC. Trimmed reads were mapped using STAR v. 2.02.01 [29] to the latest version of the melon reference genome (v.4.0) [28] (available at www.melonomics.net) and the number of reads assigned to each gene was quantified using RSEM v. 1.3.1 [107].

Two kinds of analysis were then performed. First, genes sharing similar expression profiles across all samples were obtained by performing a weighted gene co-expression network analysis, using the R package “WGCNA” v.1.69 [108]. To test for statistical differences due to the effects of genotype and treatment, a generalized linear model using the cluster’s eigengenes was performed. Kyoto Encyclopedia of Genes and Genomes (KEGG) enrichment analysis [109] of genes that were significantly enriched at adjusted *p*-value < 0.01 with each gene cluster was performed using clusterProfiler v.4.4.1 [110]. Secondly, differentially expressed genes (DEGs) were detected by using DESeq2 v.1.26.0 [30] and edgeR v.3.38.1 [31] R packages. Number of counts were normalized using the trimmed mean algorithm (TMM) as implemented in edgeR to correct for library sizes. In both cases, a linear model considering the effect of genotype (resistant (RIL-10-3) or susceptible (BO)), the effect of treatment (mock or WMV-inoculated) and its interaction at 3dpi was used. DEGs showing a significant interaction term after correction for multiple testing in DESeq2 and edgeR (adjusted p-value < 0.05) with a log2FC ≥ 1 or log2FC ≤ − 1 (log2 fold change) were considered for subsequent enrichment analysis. A consensus list of DEGs between both algorithms was obtained. DEGs between genotypes at 0 dpi were also obtained and used to check that the DEGs detected at 3 dpi were not due to genotypic differences. DEGs between resistant and susceptible genotypes were identified at 0 dpi and it was checked that those detected DEGs were not included within the list of DEGs detected for the interaction term at 3 dpi. Finally, the Cucurbits Genomics Database (CuGenDB, http://cucurbitgenomics.org/) was used to determine the enriched biological functions and processes related to the DEGs detected.

### Transcript-based single nucleotide polymorphism identification

Additionally, to identify mutations that could be related to the defense response, single nucleotide polymorphisms (SNPs) in the sequenced transcripts were detected. Clean reads were aligned to the reference melon genome by using bowtie2 v.2.3.4 [111]. and SNP calling was carried out with Freebayes v.1.3.4 [112]. Variant annotation and its predicted effect on the transcript were assayed using SNPEff v.5.0e [113].

To validate the observed genotype related to the gene *CmVps4*, a fragment of this gene was amplified by PCR in two TGR-1551, BO and RIL-10-3 cDNA samples. The corresponding PCR product was purified using the EXTRACT-ME DNA CLEAN-UP KIT (BLIRT S.A. Gdansk, Poland) and paired-end sequenced by Sanger method (Secuenciación de ADN y análisis de la expression génica, Instituto de Biología Molecular y Celular de Plantas (IBMCP), Valencia, Spain) using the primers *CmVps4P30R-F*: TCCGTCGTTCGCTTTAGTCT and *CmVPs4P30R-R*: AGTTGCAACAGCTGCATCAC.

### Validation of differentially expressed genes by RT-qPCR

To validate the RNA-seq data, the expression patterns of 6 candidate genes, putatively associated to WMV resistance in TGR-1551, were evaluated by quantitative real time PCR (qRT-PCR). Four biological and two technical replicates of both mock and WMV-inoculated plants were evaluated at 3 dpi. Total RNA (1 μg) was treated with PerfeCTa® DNase I (RNAse-free) (Quanta Biosciences, Gaithersburg, MD, United States) and reversed transcribed with RevertAid™ First Strand cDNA Synthesis Kit (ThermoFisher Scientific) and oligo (dT)20 as reverse primer. Quantitative PCR were carried out on a LightCycler480 Real-Time PCR system (Roche Applied Science, IN, USA), using the FastStart Essential DNA Green Master (Roche Molecular Systems, Rotkreuz, Switzerland) and cDNA as template. Primers sequences are listed in Additional Table 3. Melon cyclophilin (*CmCYP7*) [114] was used as reference gene. The efficiency of the primers was studied from the slope of the linear correlation between Ct and each dilution (E = 10^(−1/slope)^). To ensure specific product amplification and to avoid quantification of primer-dimers, melting curve analysis (60–95 °C) at the reaction end-point and no-template controls were used. The relative quantitative expression of each gene was calculated with the ΔCt method.

A full linear model including genotype and treatment as fixed factors and their double interaction (ΔCt ~ genotype + treatment + genotype*treatment) was used to study the observed differences in ΔCt values using the R package “stats” v.4.2.2. Normal distribution of the data was analyzed with a Shapiro-Wilk normality test, included in the R package “rstatix” v.0.7.2 [115]. As some factors combinations did not follow a normal distribution, robust two-way ANOVAs were calculated using the R package “WRS2” [116]. Finally, for the significant ANOVA test a least significant different (LSD) test, included in the R package “agricolae” v.1.3.5 [117], was performed.

### Supplementary Information

**Additional file 1.**

**Additional file 2.**

**Additional file 3.**

**Additional file 4.**

**Additional file 5.**

## Data Availability

All the data supporting our findings are contained within the manuscript, in text, tables and figures and in the supplementary files.

## Abbreviations

ABA: abscisic acid
AUX: auxins
Bg7S: basic 7S globulin-like protein
bHLH: basic helix-loop-helix
BO: “Bola de oro”
BR: brassinosteroids
bZIP: basic leucine zipper
C4H: cinnamate 4-hydroxylase
CDPK: calcium-dependent protein kinase
chr: chromosome
CK: cytokinin
CMV: cucumber mosaic virus
DEGs: differentially expressed genes
dpi: days post-inoculation
ERFs: ethylene responsive transcription factors
ET: ethylene
ETI: effector-triggered immunity
GBS: genotyping by sequencing
GCs: gene clusters
HSP: heat shock proteins
JA: jasmonate
KEGG: Kyoto Encyclopedia of Genes and Genomes
log2FC: log2 fold change
MAPK: mitogen-activated protein kinase
*MED33A*: RNA polymerase II transcription subunit 33A
MYB: myeloblastosis related
NAC: no apical meristem
NGS: new generation sequencing
NHP: N-hydroxy-pipecolic acid
PA: piperonylic acid
PAL1: phenylalanine-ammonia lyase 1
PAL2: phenylalanine-ammonia lyase 12
PAMPs: pathogen associated molecular patterns
PPP: phenylpropanoid pathway
PR: pathogenesis related
QTLs: quantitative trait loci
REF4: reduced epidermal fluorescence 4
RFR1: REF4-related 1
RILs: recombinant inbred lines
RIN: RNA Integrity Number
RLKs: receptor-like kinases
RNAPII: RNA polymerase II
ROS: reactive oxygen species
RPKs: receptor protein-kinases
SA: salicylic acid
SAR: systemic acquired resistance
SNP: single nucleotide polymorphism
TCA: citric acid cycle
TF: transcription factor
TMM: trimmed mean algorithm
ToLCNDV: Tomato leaf curl New Delhi virus
Trxs: thioredoxin-like protein
UPS: ubiquitin proteasome system
WGCNA: weighted gene co-expression network analysis
WMV: Watermelon mosaic virus
XEG: xyloglucan-specific endo-β-1,4-glucanase
ZYMV: Zucchini yellow mosaic virus
