# Supplementary material for: Insights into the early transcriptomic response against watermelon mosaic virus in melon
Source: BMC Plant Biol. 2024 Jan 20;24:58. doi: 10.1186/s12870-024-04745-x (PMC10799517; doi:10.1186/s12870-024-04745-x)
Supplement: Supplementary file 2 — Additional file 2. [file 12870_2024_4745_MOESM2_ESM.pptx]

## Slide 1
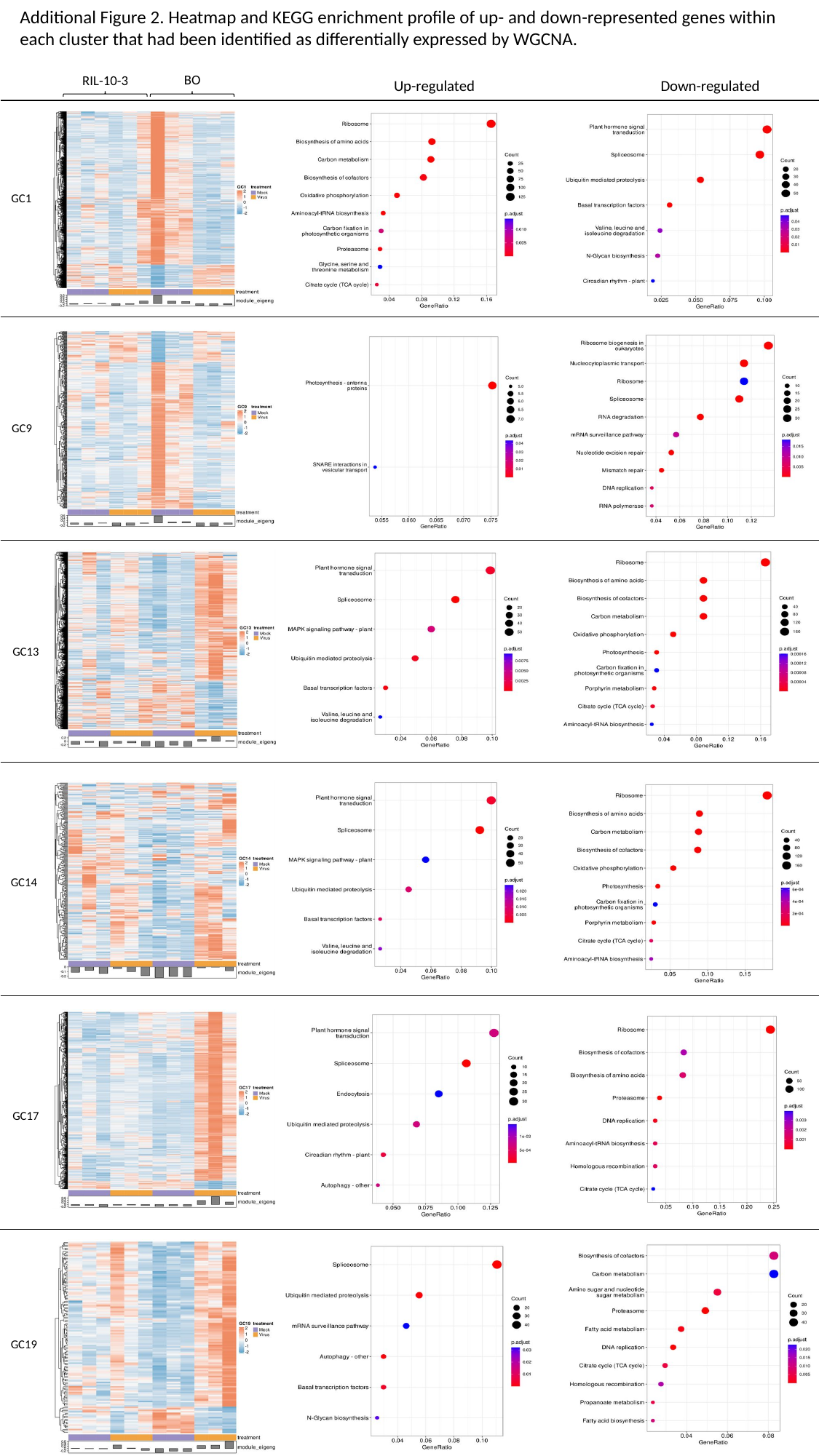

Additional Figure 2. Heatmap and KEGG enrichment profile of up- and down-represented genes within each cluster that had been identified as differentially expressed by WGCNA.
BO
RIL-10-3
Up-regulated
Down-regulated
GC1
GC9
GC13
GC14
GC17
GC19
